# Predictors of sputum culture conversion time among MDR/RR TB patients on treatment in a low-income setting

**DOI:** 10.1371/journal.pone.0277642

**Published:** 2022-11-14

**Authors:** Meiraf Daniel Meshesha

**Affiliations:** Department of Internal Medicine, Dilla University, Dilla, Ethiopia; Lady Reading Hospital, PAKISTAN

## Abstract

**Objective:**

This study aimed to assess the time to first culture conversion and its predictors among MDR/RR-TB cases enrolled in Dilchora Hospital.

**Method:**

A retrospective cohort study was conducted among MDR/RR TB cases enrolled between January 2014 and December 2018. SPSS version 26 was used for analysis. Reports are presented using percentages and frequency. Independent predictors of time-to-culture conversion were identified using multivariate Cox proportional hazard regression. Adjusted and crude hazard ratio with 95% CI was used. P-value< 0.05 declared statistical significance.

**Result:**

A total of 145 MDR/RR TB cases were included. The median time to culture conversion was at 2 months. Higher baseline hemoglobin [AHR:1.101(1.02–1.19)] and having a non-cavitary lesion on chest x-ray[AHR:1.803(1.15–2.83)] predicted a higher likelihood of early culture conversion. Resistance to at least one first-line anti-TB drug in addition to rifampicin was associated with a lower hazard of early culture conversion as compared to only rifampicin resistance[AHR: 0.577(0.37–0.91)].

**Conclusion & recommendation:**

A baseline hemoglobin level, chest x-ray finding of cavitation and resistance to rifampicin, and at least one additional drug predicted the time to culture conversion. A closer treatment monitoring and follow-up should be emphasized for those presenting with lower baseline hemoglobin, more drug resistance, and cavitation on chest x-ray.

## Introduction

Drug resistant strains of the Mycobacterium tuberculosis are among the major obstacles faced in achieving the targets of the World Health Organization(WHO) End TB Strategy by 2030 [1]. The resistance to Rifampicin (Rifampicin resistant tuberculosis/RR-TB) or to atleast Rifampicin and Isoniazid (multi-drug resistant tuberculosis/MDR-TB) is especially concerning. By 2020, WHO reported 150,359 cases of MDR/RR-TB globally initiated treatment. But the treatment outcome in these patients was favorable only in 59% of cases, according to a recent report [2].

Although there might be some variation, the second-line anti-TB drugs, which are usually given for treating RR/MDR TB, are known for being more expensive and more toxic than the first-line anti-TB drugs. They are also given for a longer period of time [3,4]. These and other factors contribute to the unsatisfactory treatment outcomes in patients treated for MDR/RR TB.

According to the WHO global lists to be used between 2016 and 2020, thirty countries account for 85% of the MDR-TB burden worldwide. Ethiopia was among the 30 high MDR-TB burden countries during this time [5]. But the country has been showing improvements regarding the identification and treatment of cases over the years. For instance, the treatment success rate for RR/MDR–TB in the county was at 70.6% in 2016. This is much higher than the WHO report for the global treatment success rate of 52% during the same year. Despite this, there remains a lot to be done [6]. The testing coverage for rifampicin resistance especially is worthy of mentioning as it remained below 80% in 2020 [2].

The time to first culture conversion is a strong predictor of treatment outcome in MDR/RR-TB patients [7–9]. Accordingly, assessing the time to first culture conversion among patients who started MDR-TB treatment is necessary. More importantly, factors that predict a prolonged time to culture conversion can be considered proxy indicators of a worse outcome for MDR/RR-TB patients on treatment. This study aims to assess the time to sputum culture conversion and its predictors among MDR/RR TB patients enrolled in Dilchora hospital MDR-TB center, Ethiopia.

## Materials and methods

### Study area and period

The study was conducted in DilChora Hospital MDR-TB treatment initiation center. It is located in Dire Dawa city administration, 501 km to the East from the capital city of Ethiopia. Since its establishment in 2013,the treatment initiation center of Dilchora hospital has served more than 150 MDR/RR-TB patients till the end of 2018.

The study took place from February 1, 2021 –May 31, 2021.

### Study design

A hospital-based, retrospective cohort study was carried out.

### Study population

All cases of MDR/RR-TB that initiated treatment between January 2014 and December 2018 in the treatment initiation center of Dilchora Hospital.

### Inclusion criteria

All cases of MDR/RR-TB, that initiated treatment between January 2014 and December 2018 in the treatment initiation center of Dilchora Hospital and having a complete data on outcome variable were included.

### Exclusion criteria

Those cases with incomplete data on the outcome variable were excluded from the study. For this reason, one patient was excluded from the study and the rest 145 were included.

### Diagnostic techniques

Patients with MDR/RR TB were identified using a GeneXpert MTB/RIF assay and then sputum samples were taken for culture and drug sensitivity test (DST). After initiation of treatment, sputum samples were taken monthly so that experienced technicians would evaluate the sputum smear and culture.

Evaluation and documentation of chest X-rays readings were performed by radiologists.

### Treatment of MDR/RR-TB

The treatment regimens used include a standardized regimen and individualized regimens. Each of these treatment regimens were given as per the national guidelines on drug-resistant TB [10].

At initiation of treatment for MDR/RR TB, standardized regimen was given for all patients. A change to an individualized regimen would occur after the DST results arrive and a strain with fluoroquinolones/second-line injectables resistance was detected.

The standardized treatment regimen consists levofloxacin, kanamycin/amikacin, cycloserine, ethionamide, pyrazinamide and ethambutol. But the individualized regimen was guided by the DST results. The direct observation treatments(DOTs) was implemented for both regimens.

### Data collection procedure

A structured questionnaire was used to collect data from treatment charts, laboratory and radiologic reports.

### Data quality control

Intensive training was given for data collectors and supervisors before onset of data collection process. A daily inspection of data completeness and consistency was performed by the supervisors and principal investigator. Double data entry and validation using Epi- Data version 3.1 was also done to assure quality of data.

### Data analysis

SPSS version 26 was used for analysis. For categorical variables, frequency and percentage were used, while mean±SD or median±IQR were used for continuous variables. The outcome variable was dichotomized into early culture conversion versus censored for survival analysis.

Factors related to early culture conversion were analyzed using the Cox proportional hazard model. Hazard ratio and adjusted hazard ratio with 95% confidence interval were used to assess the variables. A graphic check and verification using log-log plots was performed to assess if proportional hazards assumption required to use Cox proportional hazard model was fulfilled.

To control the confounding effect of variables, multivariate Cox proportional hazard regression was used. Variables with p value less than or equal to 0.25 on bivariate analysis were used as candidates for multivariable analysis. A statistically significant association was declared when P value was less than 0.05 on multivariate analysis.

### Operational definition

**Cured:** During the final 12 months of treatments, MDR/RR-TB patient with at least 5 consecutive negative cultures where the samples were collected with a gap of atleast 30 days [10].

**Early sputum culture conversion**: A first sputum culture conversion from positive to negative after taking a maximum two months of MDR-TB treatment.

**Delayed sputum culture conversion**: A first sputum culture conversion from positive to negative after taking more than two months of MDR-TB treatment.

**Died**: A death that occurred for any reason during the treatment course [10].

**Failure**:During the final 12 months of therapy, if 2 or more of 5 cultures were positive or if any of the final 3 cultures is positive.

**Individualized Treatment**: A regimen adjusted considering the patient’s prior history of TB treatment, DST results and possible side effects [10].

**Loss-to-follow-up:** Anti-TB treatment interruption for 2 or more consecutive months without medical approval [10].

**MDR-TB**: Tuberculosis caused by a strain resistant to at least isoniazid and rifampicin

**RR-TB:** Tuberculosis caused by a rifampicin-resistant without isoniazid resistance

**Serum creatinine**: Value less than 1.2 mg/dl was considered normal, while value ≥1,2mg/dl was considered abnormal [11].

**Standardized treatment**: A regimen that is based on data from representative patient populations in the absence of individual DST [10].

**Treatment completed**: Completion of treatment as per the national treatment protocol but failing to fulfil the definition of cure for absent bacteriological results [10].

**Treatment delay**: The time period between the MDR/RR-TB diagnosis confirmation and treatment initiation

**Treatment Failure**: During the final 12 months of therapy for MDR/RR-TB, if 2 or more of 5 cultures are positive or if any 1 of the final 3 cultures is positive [10].

### Dependent and independent variables

#### Dependent variable

Time to culture conversion; Early/ Delayed

#### Independent variables

Age, sex, hemoglobin, WBC count, BMI, baseline creatinine, HIV status, anti-TB exposure, treatment delay, drug sensitivity test, radiologic pattern, presence of ADR, and comorbidity other than HIV

A treatment delay of 60 days or more after diagnosis was considered a significant delay in MDR-TB treatment initiation [12].

### Ethical statement

Dire Dawa Regional Health Bureau and Dil-Chora hospital gave the ethical approval for conducting this study. As the data was accessed from patients’ charts(secondary data), participant consent was not applicable. But the confidentiality and privacy of the patients was strictly kept throughout the process. I confirm that this study complies with the Declaration of Helsinki.

## Results

A total of 146 patients were enrolled into the center during the study period, of which 145 had a complete data on outcome variable and were included in this study.

### Socio-demographic characteristics of the patients

In this study, most of the patients were below the age of 40 years (112, 77.2%). The mean age was 29.6 (±12.4) years. There were more males (86, 59.3%) than females (59, 40.7%) (Table 1).

**Table 1:** Socio-demographic characteristics of MDR/RR-TB patients in Dilchora Hospital, Dire Dawa, Eastern Ethiopia.

Characteristics	Early sputum culture conversion (≤2 month)	Delayed sputum culture conversion(>2 month)	Chi-Square P-value
**Age** (mean±SD = 29.6 ±12.4)			
<40	77(68.7%)	35(31.3%)	0.662
≥40	24(72.7%)	9(27.3%)	
**Sex**			
Male	65(75.6%)	21(24.4%)	0.061
Female	36(61.0%)	23(39.0%)	

**Fig 1:**
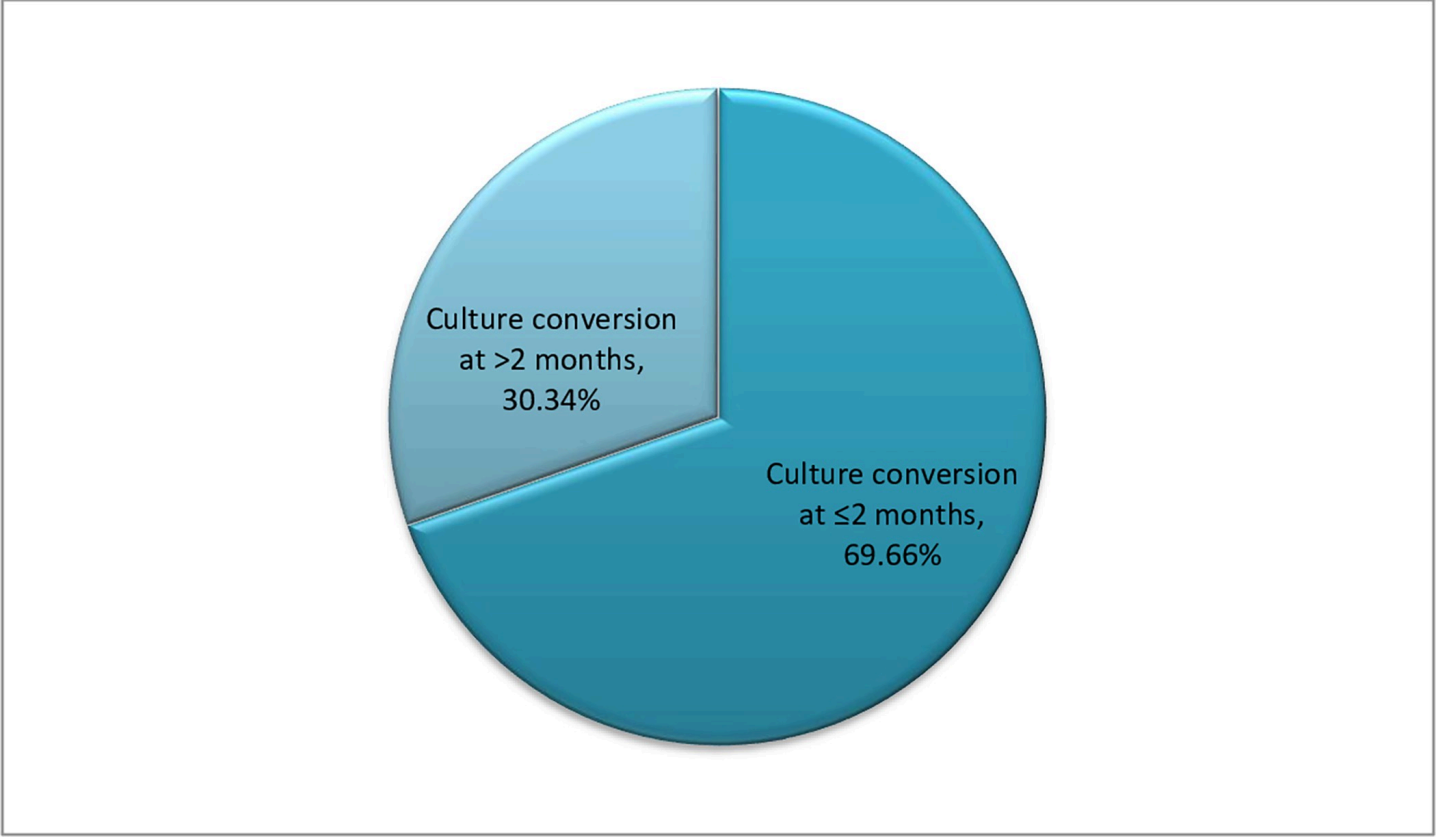
Time of culture conversion of MDR/RR TB patients who were on treatment in Dilchora Hospital, Eastern Ethiopia (n = 145), 2014–2018.

A larger proportion of the patients, 101(69.7%), had a sputum culture conversion at the 2^nd^ month of treatment or prior to that (Fig 1). A similar trend of higher culture conversion within 2 month of treatment initiation was also seen in the different age and sex categories (Table 1).

### Clinical characteristics of the patients

Regarding the clinical characteristics of our patients, there was a 17.9% HIV positivity rate in the participants. About 37(25.5%) of the patients had some form of comorbidity other than HIV, including diabetes, hypertension, COPD,cardiac illness and others. The body mass index was below 18.5kg/m^2^ in 108(74.5%) patients, while only 37 patients had a BMI≥18.5 kg/m^2^ (Table 2).

**Table 2:** Clinical characteristics of MDR/RR-TB patients in Dilchora Hospital, Dire Dawa, Eastern Ethiopia.

Characteristics	Early sputum culture conversion(≤2 month)	Delayed sputum culture conversion(>2 month)	Chi-square test P-value
**HIV status**			
Negative	86(72.3%)	33(27.7%)	0.143
Positive	15(57.7%)	11(42.3%)	
**Comorbidity**			
Yes	28(75.7%)	9(24.3%)	0.356
No	73(67.6%)	35(32.4%)	
**BMI**			
<18.5	72(66.7%)	36(33.3%)	0.181
≥18.5	29(78.4%)	8(21.6%)	
**Hemoglobin(g/dl)**			
<10	30(60%)	20(40%)	0.067
≥10	71(74.7)	24(25.3%)	
**Serum creatinine**			
<1.2	95	42	0.731(G^2^)
≥1.2	6	2	
**Previous exposure to anti-TB (1**^**st**^ **or 2**^**nd**^ **line)**			
Yes	79(66.4%)	40(33.6%)	0.067
No	22(84.6%)	4(15.4%)	
**Treatment delay**			
<60 days	78(70.9%)	32(29.1%)	0.365
≥60 days	20(62.5%)	12(37.5%)	
**Drug resistance pattern**			
Rifampicin only	62(81.6%)	14(18.4%)	0.001
Rifampicin and at least 1 additional drug	39(56.5%)	30(43.5%)	
**Radiologic pattern on Chest X-ray**			
Cavitary	34(58.6%)	24(41.4%)	0.018
Non-Cavitary	67(77.0%)	20(23.0%)	
**ADR to MDR-TB treatment**			
Yes	89(69.0%)	40(31.0%)	0.616(G^2^)
No	12(75.0%)	4(25.0%)	
**Final treatment outcome**			
Cured	83(78.3)	23(21.7%)	0.0001(G^2^)
Completed	11(50.0%)	11(50.0%)	
Died	3(25.0%)	9(75.0%)	
Lost-to-follow up	4(80.0%)	1(20.0%)	

Only 26(17.9%) patients had previously had no exposure to any form of anti-TB. The drug resistance pattern shows about 52.4% of the patients having resistance to Rifampicin only, while the rest had resistance to Rifampicin and at least one additional first-line anti TB (Table 2).

The radiologic pattern on chest x ray was cavitary in the minority of the patients (58, 40%), but there was a statisticaly significant association with the time to culture conversion. Majority(141) of the patients were started on standardized regimen for MDR-TB while only 4 patients received the individualized protocol. Adverse drug reaction to the MDR TB regimen was seen in most patients (129, 89.0%). Analyzing the final treatment outcome, there were 12 deaths and 5 patients were lost from follow up (Table 2).

In most of the clinical characteristics’ variables, there was a higher tendency for the patients to have a culture conversion at 2 month or prior to that across the different categories. The exception was those with an outcome of death where a higher proportion of culture conversion was seen after the 2^nd^ month (75%). The association was statistically significant(P-value:0.0001) (Table 2).

### The time to first sputum culture conversion of MDR/RR- TB patients

**Fig 2:**
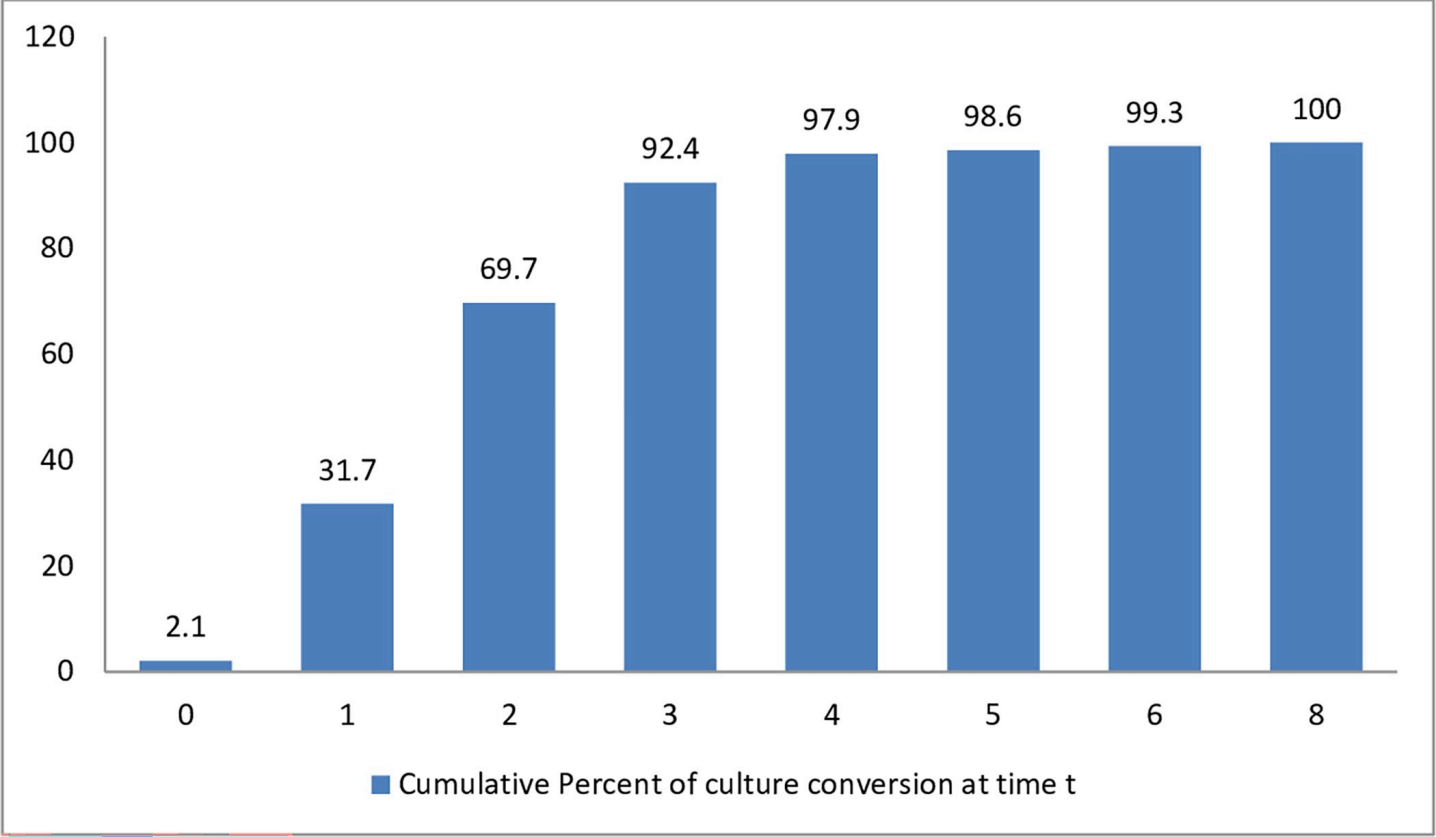
Time to sputum culture convesion in months for MDR/RR TB patients who were on treatment at Dilchora Hospital, Eastern Ethiopia (n = 145).

The total follow-up time for this cohort was 304.5 person-months.The median time to first culture conversion was at 2 months(IQR:1–3). About 69.7% (101) of the patients had their first culture conversion by the time they finished the 2^nd^ month of treatment. By the end of the 4 months, 97.9% of the patients had culture conversion. The last person to have a delayed culture conversion had it at the end of the 8^th^ month of treatment (Fig 2).

### Bivariate and multivariate analysis

Bivariate Cox regression analysis was run for the variables of sex, age, BMI(kg/m2), hemoglobin(g/dl), WBC, baseline creatinine, Anti-TB exposure (1st or 2nd line), HIV status, comorbidity other than HIV, drug sensitivity test, radiologic pattern, treatment delay, and Presence of ADR. The model was adequately powered.

On bivariate analysis: sex, baseline hemoglobin level, baseline WBC count, anti TB exposure, drug sensitivity test, and radiologic pattern were found to have a P-value<0.25 and became candidates for multivariable analysis.

On multivariable analysis, three independent variables were found to be significant predictors of early culture conversion. For every 1gm/dl increment of the baseline hemoglobin, the hazards of early culture conversion increased by 10.1% [AHR: 1.101(1.02–1.19)]. The likelihood of having an early culture conversion was also 1.8 times higher for those patients with a non-cavitary lesion on chest x-ray than those with a cavitary lesion [AHR:1.803(1.15–2.83)]. On the other hand, patients who had resistance to rifampicin and at least 1 additional first-line anti TB drug had a 42.3% lower hazard of having an early culture conversion than those who had resistance to only rifampicin [AHR: 0.577(0.37–0.91)] (Table 3).

**Table 3:** Multivariate analysis of variables showing the predictors of time to culture conversion among MDR/RR TB patients following treatment in Dilchora Hospital, Dire Dawa, Eastern Ethiopia (n = 145).

Variable	category	AHR	95% CI	P-value
Sex	Male	1	-	0.058
	Female	0.646	0.41–1.02	
Baseline Hemoglobin(g/dl)	Median(IQR) 11.1(9.5–12.9)	1.101	1.02–1.19	**0.011**
Baseline WBC(× 10^9^/L)	Median(IQR) 7.5(5.2–9.4)	0.962	0.91–1.02	0.224
Anti-TB drug exposure	No	1	-	0.655
	Yes	0.880	0.50–1.54	
Drug sensitivity test	Rif only resistance	1	-	**0.017**
	Rifampicin+ at least 1 other	0.577	0.37–0.91	
Radiologic pattern	Cavitary	1	-	**0.010**
	Non-cavitary	1.803	1.15–2.83	

Post-hoc power analysis was done using STATA 15.0 version for the drug sensitivity test variable using a 0.05 significance level, sample size of 145 and Hazard ratio of 0.577. It showed that the study had a 91% power for detection of differences in culture conversion time between the different categories of drug resistance pattern (rifampicin only versus rifampicin plus atleast one additional drug resistance).

**Fig 3:**
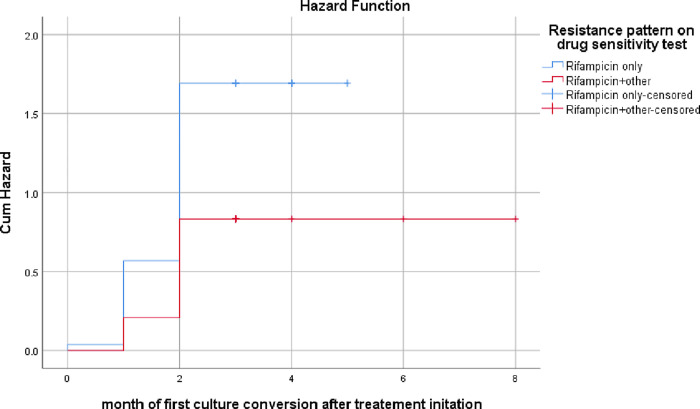
Hazard function for early conversion depending on drug resistance pattern among MDR/RR TB patients who were on treatment at Dilchora Hospital, Eastern Ethiopia.

As can be seen clearly from the Hazard function graph (Fig 3), patients with drug resistance to only rifampicin were more likely to have an early culture conversion than those with more drug resistance.

**Fig 4:**
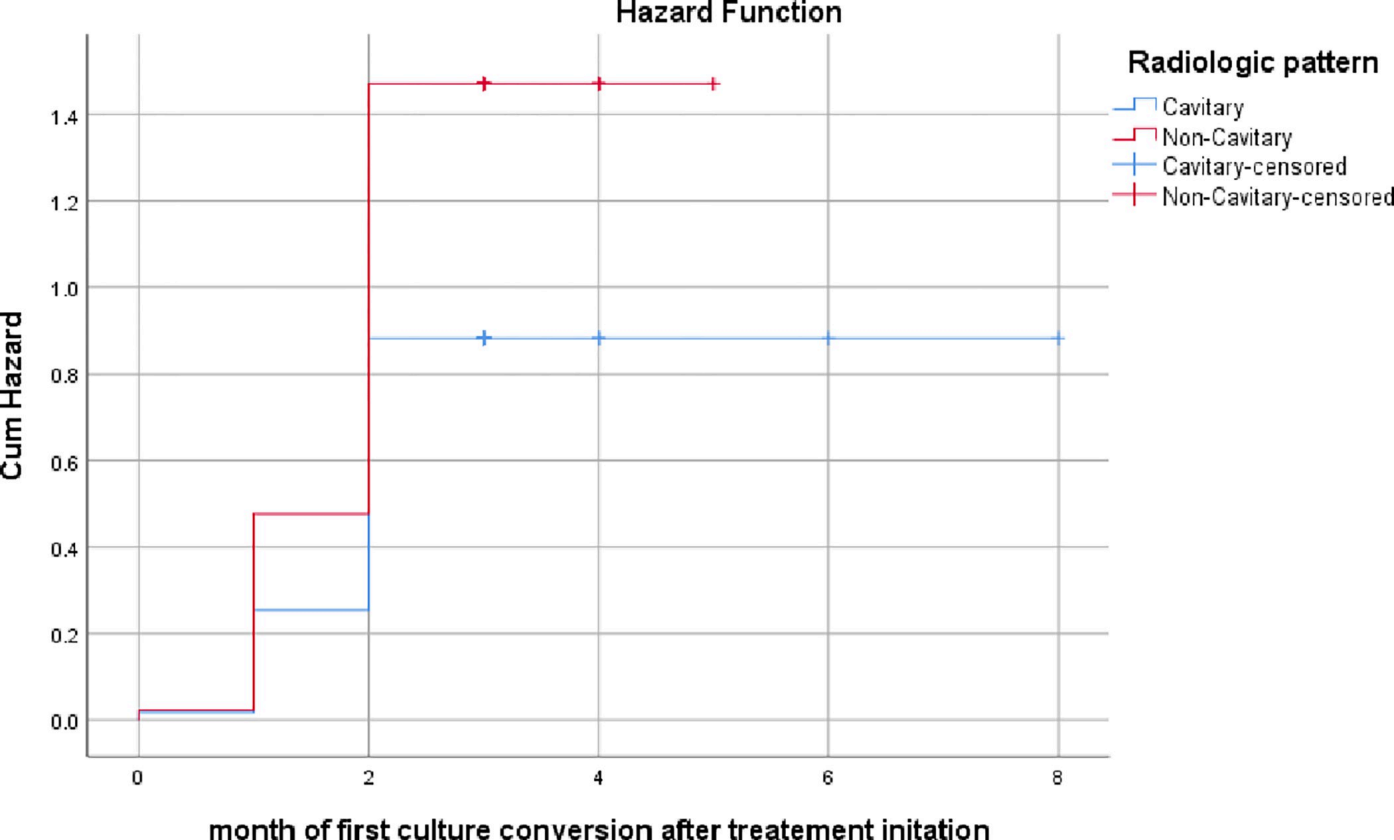
Hazard function for early conversion depending on radiologic pattern among MDR/RR TB patients who were on treatment at Dilchora Hospital, Eastern Ethiopia.

Similarly, patients with non-cavitary lesion on chest x-ray had a higher likelihood of an early culture conversion than those with a cavitary lesion (Fig 4).

## Discussion

This study tried to assess the time for first culture conversion and its predictors among MDR/RR TB patients treated at Dil-chora Hospital, Eastern Ethiopia. The median time to first culture conversion was at 2 month. Base line hemoglobin, drug sensitivity test, and radiologic finding were found to be independent predictors of culture conversion in MDR-TB cases.

Overall, less than one-thirds of the patients had a culture conversion after the second month of treatment. The findings of this study also indicate that patients with a higher hemoglobin level are more likely to have a rapid culture conversion. On the other hand, a non-cavitary lesion on chest x ray and a drug resistance to rifampicin and at least one additional second-line drug were predictors of a delayed culture conversion.

In our study, the median time to culture conversion was found to be at 2 month. Similar finding were also seen in studies coming from Peru(59 days) [13], Lativia(60 days) [8] and Indonesia(2 months) [14]. But there were also reports with either a shorter conversion time, like the study from Japan which had reported a 39 day median time of conversion [15], or a much longer conversion time(159 days) as seen from a report in China [16]. The differences in the median time may be due to the difference in the socio-demographic, clinical characteristics, genetics and treatment regimen used among the participants of these studies. The findings from recent studies in Ethiopia also support this hypothesis. The median time to culture conversion from Oromia region of Ethiopia was reported to be 61 days [17] while it was reported to be 65 days in the Amhara region of the country [18]. Similarly, a metanalysis that included 9 articles from East Africa revealed the median time sputum culture conversion to 61.2 days [19].

The current guidelines recommend that monitoring of MDR/RR TB treatment should include a monthly follow-up of sputum culture conversion [20]. A report from a study of the association between time of culture conversion and the final outcome showed that patients who had failed to achieve culture conversion within 2 months were more likely to have worse outcomes [8]. Our study also found that, of all the patients who eventually died, about 75% of them had a delayed culture conversion. This finding highlights the importance of the time to sputum culture conversion and its implication on the outcome of patients on MDR/RR-TB treatment. A delayed culture conversion might indicate a sub-optimal therapeutic effect of the drugs which can be due to various reasons. The possible reasons include inherent resistance of the bacillary strain, improper administration of drugs, poor penetration of drugs into cavities, or a host’s poor immunity. But, whatever the case, the patient would likely be going through a prolonged period of illness with possible widespread dissemination of the bacilli eventually leading to death.

The presence of cavitary lesions on chest x-ray was also a strong predictor of delayed culture conversion in our study. This may be due to a difficulty in penetration of the drugs into these cavities and there by reducing the anti-bactericidal activity which eventually prolongs the time to culture conversion. Many other studies from different areas also found a similar result [8,21,22].

The other important predictor of delayed culture conversion in our study was drug resistance to rifampicin and at least one additional drug. This was also illustrated from studies in US [23], Nigeria [24] and Lativia [8], where resistance to more drugs was associated with a prolonged time of culture conversion. This finding, in addition to predicting time to culture conversion, helps in guiding proper antibiotic regimens. Nevertheless, resistance to more drugs would eventually limit our options and predispose patients to a more toxic and less effective regimen.

The effect of hemoglobin level in the prediction of time to culture conversion has not been well studied. Our study, however, found that a higher baseline level of blood hemoglobin predicts a shorter time of culture conversion. On the one hand, this effect can be explained by the fact that a lower hemoglobin level might be a surrogate marker of disease severity [25], and it is only logical to assume that a severe disease most likely prolongs the time to culture conversion. On the other hand, anemia, more specifically Iron deficiency anemia is associated with abnormal immune functions like altered humoral, phagocytic, and IL 6 functions [26]. The altered phagocytic functions of macrophages and IL 6 as a result of anemia in turn result in increased disease severity [27] and subsequently delayed culture conversion.

### Strength and limitation of the study

The strength of this study is that it tried to assess the time it takes for culture conversion in MDR/RR TB patients on treatment, giving highlights for the practicing physician when to be concerned especially in a resource-limited setup. Additionally, it included factors like baseline hemoglobin which were not addressed in previous studies and were found to be predictors of delayed conversion. This might also help in prioritizing high-risk patients and giving due attention.

The relatively small sample size might have limited the power of the study. The exclusion of a case with missing data on outcome variable might also have had a slight effect on the result. The fact that data was collected retrospectively from secondary sources might also have an effect the quality of the study.

## Conclusion

Our study found that the median time to culture conversion in MDR/RR TB patients on treatment was 2 months. The factors which had predicted a delayed time to culture conversion were having a cavitary lesion on chest x ray, bacilli resistance to rifampicin and at least 1 additional anti-TB drugs and lower baseline hemoglobin level.

A close follow-up of patients who show resistance to more than 1 anti-TB drugs and adjusting our choice of second-line treatment based on drug sensitivity is crucial. Additionally, patients with a low baseline hemoglobin level should be given more emphasis with early identification and proper treatment of the condition, as it can affect the treatment outcome.

## Supporting information

S1 File (SAV)
